# 2135 Impact of primary care physician gatekeeping on medication prescriptions for atrial fibrillation

**DOI:** 10.1017/cts.2018.287

**Published:** 2018-11-21

**Authors:** Andrew Y. Chang, Mariam Askari, Jun Fan, Paul A. Heidenreich, P. Michael Ho, Kenneth W. Mahaffey, Alexander C. Perino, Mintu P. Turakhia

**Affiliations:** 1 Veterans Affairs Palo Alto; 2 Stanford University School of Medicine; 3 University of Colorado at Denver

## Abstract

OBJECTIVES/SPECIFIC AIMS: Atrial fibrillation (AF) is the most commonly encountered arrhythmia in clinical practice, and has widely varying treatments for stroke prevention and rhythm management. Some of these therapies are increasingly being prescribed by primary care physicians (PCPs). We therefore sought to investigate if healthcare plans with PCP gatekeeping for access to specialists are associated with different pharmacologic treatment strategies for the disease. In particular, we focused on oral anticoagulants (OACs), non-vitamin K-dependent oral anticoagulants (NOACs), rate control, and rhythm control medications. METHODS/STUDY POPULATION: We examined a commercial pharmaceutical claims database (Truven Marketscan™) to compare the prescription frequency of OAC, rate control, and rhythm control medications used to treat AF between patients with PCP-gated health plans (where the PCP is the gatekeeper to specialist referral—i.e., HMO, EPO, POS) and patients with non-PCP-gatekeeper health plans (i.e., comprehensive, PPO, CHDP, HDHP). To control for potential confounders, we also used multivariable logistic regression models to calculate adjusted odds ratios which accounted for age, sex, region, Charlson comorbidity index, CHADS2Vasc score, hypertension, diabetes, stroke/transient ischemic attack, prior myocardial infarction, peripheral artery disease, and antiplatelet medication use. We also calculated median time to therapy to determine if there was a difference in time to new prescription of these medications. RESULTS/ANTICIPATED RESULTS: We found only small differences between patients in PCP-gated and non-PCP-gated plans regarding prescription proportion of anticoagulants at 90 days following new AF diagnosis (OAC 44.2% vs. 42%, *p*<0.01; warfarin 39.1% vs. 37.1%, *p*<0.01; NOAC 5.9% vs. 6.0%, *p*=0.64). We observed similar trends for rate control agents (76.4% vs. 73.4%, *p*<0.01) and rhythm control agents (24.4% vs. 24.6%, *p*=0.83). We found similar odds of OAC prescription at 90 days following new AF diagnosis between patients in PCP-gated and non-PCP-gated plans (adjusted OR for PCP-gated plans relative to non-gated plans: OAC 1.006, *p*=0.84; warfarin 1.054, *p*=0.08; NOAC 0.815, *p*=0.001; dabigatran 0.833, *p*=0.004; and rivaroxaban 0.181, *p*=0.02). We observed similar trends for rate control agents (1.166, *p*<0.0001) and rhythm control agents (0.927, *p*=0.03). Elapsed time until receipt of medication was similar between PCP-gated and non-gated groups [OAC 4±14 days (interquartile range) vs. 5±16 days, *p*<0.0001; warfarin 4±14 vs. 5±14, *p*<0.0001; NOAC 7±26 vs. 6±23, *p*=0.2937; rhythm control 13±35 vs. 13±34, *p*=0.8661; rate control 10±25 vs. 11±30, *p*<0.0001]. DISCUSSION/SIGNIFICANCE OF IMPACT: We found that plans with PCP gatekeeping to specialist referrals were not associated with clinically meaningful differences in prescription rates or delays in time to prescription of oral anticoagulation, rate control, and rhythm control drug therapy. In some cases, PCP gatekeeping plans had very small but statistically significant lower odds of being prescribed NOACs. These findings suggest that PCP gatekeeping does not appear to be a major structural barrier in receipt of medications for AF, although non-PCP-gated plans may vary slightly favor facilitating the prescription of NOACs. Our findings that overall OAC prescriptions did not differ by PCP gating status may suggest completion of the rapid dissemination and uptake phase for most AF treatments. The small but statistically significant odds ratios favoring the non-PCP-gated populations in NOACs further suggests that in this newer drug group, the process is ongoing, with more specialists representing early adopters. Interestingly, the low primary care odds ratio of rivaroxaban use, relative to dabigatran, may be indicative of a gradient of uptake of later-generation NOACs, although interpretability is limited by the small number of patients in the rivaroxaban group.

